# Noroviruses in Archival Samples

**DOI:** 10.3201/eid1103.040838

**Published:** 2005-03

**Authors:** Sylvain Skraber, Ronald Italiaander, Willemijn J. Lodder, Ana Maria de Roda Husman

**Affiliations:** * National Institute for Public Health and the Environment, Bilthoven, The Netherlands

**Keywords:** dispatch, norovirus, RT-PCR detection, sewage, water, effluent, storage, retrospective study, archival samples

## Abstract

Application of recent techniques to detect current pathogens in archival effluent samples collected and concentrated in 1987 lead to the characterization of norovirus GGII.6 Seacroft, unrecognized until 1990 in a clinical sample. Retrospective studies will likely increase our knowledge about waterborne transmission of emerging pathogens.

Noroviruses, previously designated as small round-structured viruses or Norwalk-like caliciviruses, are enteric viruses that cause large outbreaks of gastroenteritis (1). Besides person-to-person transmission, these viruses may spread by water. Noroviruses cannot be propagated by cell culture (2), and detecting them by using immunologic or electron microscopic techniques is painstaking and time-consuming. When molecular techniques were developed in the early 1990s, norovirus detection in water and subsequent genotyping became feasible (3). Noroviruses are, therefore, more frequently identified as the causative agent in waterborne outbreaks (1,4). Though humans are frequently infected with 1 specific norovirus strain, many different strains are found in sewage and surface water (5). Based on the comparison of open reading frame 2 sequences, GGI and GGII comprise 5 and 10 genotypes, respectively; all are associated with infections in humans (6). Recently, other regions have been used for norovirus classification, such as the capsid VP1 region, leading to 7 GGI and 12 GGII genotypes (7). This classification may evolve further, as a recent study proposed to define 3 new human genogroups, IV, VI, and VII (8). Season-novel variants may have characteristics that enable them to replace the predominant strain circulating in the population (4). Primer pairs and probes used in norovirus detection need to be optimized to include such novel strains (9). In that context, previously screened water samples may have been falsely negative, or some noroviruses may have been missed.

## The Study

We conducted a retrospective study on 4 archival effluent samples collected and concentrated in 1987, analyzed for phages and enteroviruses but not noroviruses and kept frozen at –70°C. We analyzed these samples for noroviruses in 2003 by using JV12Y and JV13I, a recently optimized primer set that allows detection of a broad range of noroviruses by targeting the RNA-dependent RNA polymerase (9).

A sample of effluent waters from the sewage treatment plant situated in Leerdam, the Netherlands, was taken on July 22, August 5, August 26, and September 9, 1987. Each of the 4 samples was concentrated by using a conventional filter adsorption-elution method (10), and the resulting eluates were reconcentrated by ultrafiltration. The 4 ultrafiltrates were analyzed for somatic phages, F-specific phages, and enteroviruses, and each sample was found positive for these viruses. Samples were stored at –70°C. A norovirus reverse transcription–polymerase chain reaction (RT-PCR)-positive stool sample, obtained in 1997 and kept at 4°C, was used as a positive control for cloning and sequencing.

The RT-PCR was conducted as described previously (5). Briefly, 7-mL effluent samples were clarified by centrifugation for 10 min at 3,000 *g*, whereas 10 µL of stool sample was diluted in 3 mL of sterile water. RNA was extracted from the resulting supernatant of the effluent sample and the total volume of diluted stool sample by binding to silica beads in the presence of guanidinium isothiocyanate (11). Five microliters of the extracted RNA was reverse transcribed for 60 min at 42°C after annealing with JV13I (9) at 0.3 µmol/mL in 15 µL of 10 mmol Tris–HCl pH 8.3, 50 mmol KCl, 3 mmol MgCl_2_, 1 mmol deoxynucleoside triphosphate, 40 U/mL RNAguard, and 5 U AMV-RT (Promega, Leiden, the Netherlands). Five microliters of the RT mix was added to 45 µL of a PCR-mix containing 10 mmol Tris–HCl pH 9.2, 50 mmol KCl, 1.2 mmol MgCl_2_ (final concentration 1.5 mmol), 0.2 mmol dNTPs, 2.5 U ampliTaq, and 0.3 µmol/mL of JV12Y (9). Samples were denatured for 3 min at 94°C and subjected to 40 cycles (94°C for 1 min, 37°C for 1 min 30 s, and 74°C for 1 min) before linearization at 74°C for 7 min. Amplified DNA was detected by electrophoresis in a 2% agarose gel and visualized under blue light after SYBR-Gold (nucleic acid gel stain) (Molecular Probes, Leiden, the Netherlands) staining. The specificity of the detected noroviruses was confirmed by Southern blot hybridization as described previously (5). RT-PCR products of appropriate size (327 bp) were gel purified (QIAquick PCR purification kit, Qiagen, Hilden, Germany) and cloned into a plasmid vector (pGEM-T Easy Vector, Invitrogen, Leek, the Netherlands). Plasmid DNA was purified and amplified by PCR using specific plasmid M13 forward and reverse primers according to manufacturer instructions. Amplified DNA was confirmed to be norovirus specific by Southern blot hybridization (using the same protocol as described above) before sequencing using the BigDye Terminator Cycle Sequencing Ready reaction Kit (PE Applied Biosystems, Foster City, CA, USA). Multiple sequence alignments were performed on the 145 sequenced bases with sequences of known genetic clusters available from GenBank, and phylogenetic trees were generated by using Bionumerics software (V2.0 Applied Maths, Kortrijk, Belgium).

Amplification of RNA detected from the stool sample and 3 of the 4 effluent water samples from 1987 yielded a norovirus-specific 327-bp band after gel electrophoresis of the RT-PCR product (data not shown). The presence of norovirus was confirmed by Southern blot hybridization of the amplified cDNA. RT-PCR products derived from the stool sample and 1 effluent water sample were successfully cloned and sequenced. The multiple sequence alignment and the resulting phylogenetic tree (Figure) showed high similarity between norovirus amplified from stool and the GGII.4 Hu/NLV/Grimsby/95/UK strain (GenBank accession no. AJ004864) (score: 143/145 nt). In the same way, high similarity was found between norovirus amplified from effluent and the GGII.6 Hu/NLV/Seacroft/1990/UK (GenBank accession no. AJ277620) (score: 144/145 nt). Results were not likely due to contamination, as the stool sample was positive for a norovirus strain different from the effluent sample, and the negative controls for RNA extraction and RT-PCR were negative (data not shown).

**Figure:**
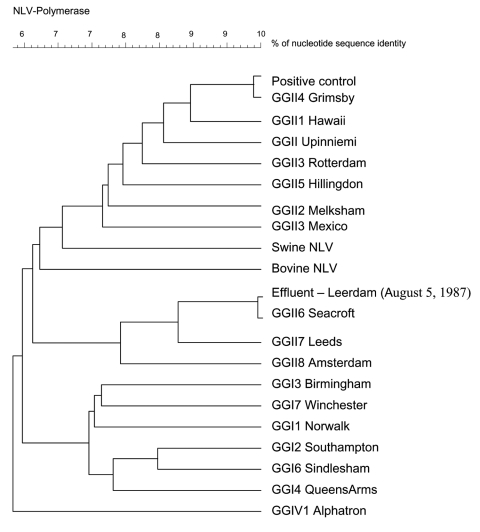
Phylogenetic analysis of the positive stool sample, the 1987 effluent sample, and referenced norovirus strains based on 145 nt of the RNA-dependent RNA polymerase sequence.

## Conclusions

Historically, Seacroft strain was first detected and sequenced from a stool sample collected in 1990 in the United Kingdom (12). Noroviruses are generally more easily detected in clinical samples in which the virus concentrations are higher. Furthermore, norovirus strains present important genetic variations that can explain commonly reported, false-negative RT-PCR results (9,13). For those reasons, norovirus prevalence may be underestimated, especially in environmental samples in which virus concentrations are low and RT-PCR inhibition may occur. Their detection in stool samples enables optimization of primers that can subsequently be used to screen water samples. In that context, our observation confirms, retrospectively, the potential usefulness of environmental surveillance as a tool for monitoring virus infections in the population. Indeed, our results show that Seacroft strain had already spread in the environment at least 3 years before its reported characterization from a clinical sample. Moreover, this strain has been detected in the middle of summer (August 5, 1987), which confirms that norovirus infections do not exclusively occur during winter (4,14). Finally, our results show that environmental archival samples stored at low temperature with beef extract as cryoprotector may profit from current virologic detection methods. Thus, retrospective studies may provide information about geographic and seasonal distribution of emerging or previously undetectable viral strains. Forthcoming virus detection methods may provide useful information about current environmental samples. For example, no method is available to ascertain the presence of infectious norovirus and such methodology should be developed (2). We confirmed the presence of infectious F-specific phages and somatic coliphages in all 4 archival samples after 17 years of storage at –70°C, following ISO/FDIS 10705-1 and 10705-2 protocol, respectively (data not shown). We also cultured enteroviruses on buffalo green monkey cells (BGM) and detect plaques by monolayer BGM plaque assay (data not shown). Similar counts were established for the enteroviruses in 1987 (1–22 PFU/g of concentrate) and in 2004 (0.5–29 PFU/g of concentrate). Therefore, if frozen concentrates also conserve the integrity of norovirus, new detection protocols may help to identify infectious noroviruses in the environment. From a methodologic point of view, long-term retrospective virologic studies based on screening of archival samples have 2 advantages: 1) using the same methodology to generate results allows easier comparison, and 2) it can be applied to many samples already collected over a period of years. When this approach is used, important knowledge on pathogenesis and disease progression in clinical settings has already been acquired (15).

Moreover, environmental samples potentially differ from clinical samples in 2 important ways. First, environmental samples consist of pathogenic viruses derived from different persons that represent large populations, whereas clinical samples represent single persons. Therefore, environmental samples potentially contain more variant strains. Indeed, in environmental samples, both symptomatic and asymptomatic patients contribute to the dissemination of virus strains. These strains that can multiply in their host without causing disease are neglected when analyzing clinical samples, which are usually collected from patients with acute gastroenteritis symptoms. Second, viruses that are discharged in the environment through contaminated wastewater are subjected to diverse physical, chemical, and biologic inactivation or degradation factors (e.g., sunlight, wastewater treatment). These factors favor selection of the most persistent variant strains in the environment. Therefore, these strains have a higher probability of reaching and infecting persons through waterborne transmission. In that context, environmental samples may be considered a source of information about emerging waterborne viruses.

In conclusion, using long-term retrospective studies to analyze stored environmental and clinical samples may be a promising way of increasing our knowledge about the emergence of novel pathogens in waterborne disease transmission.

This study was financially supported by the Environmental Inspectorate project number 330000, Health Related Water Microbiology.
